# Doxycycline potentiates antitumor effect of 5-aminolevulinic acid-mediated photodynamic therapy in malignant peripheral nerve sheath tumor cells

**DOI:** 10.1371/journal.pone.0178493

**Published:** 2017-05-30

**Authors:** Ming-Jen Lee, Shih-Hsuan Hung, Mu-Ching Huang, Tsuimin Tsai, Chin-Tin Chen

**Affiliations:** 1 Department of Neurology, National Taiwan University Hospital, Taipei, Taiwan; 2 Department of Medical Genetics, National Taiwan University Hospital, Taipei, Taiwan; 3 Department of Biochemical Science and Technology, National Taiwan University, Taipei, Taiwan; 4 Graduate Institute of Biomedical Materials and Tissue Engineering, College of Oral Medicine, Taipei Medical University, Taipei, Taiwan; Massachusetts General Hospital, UNITED STATES

## Abstract

Neurofibromatosis type 1 (NF1) is one of the most common neurocutaneous disorders. Some NF1 patients develop benign large plexiform neurofibroma(s) at birth, which can then transform into a malignant peripheral nerve sheath tumor (MPNST). There is no curative treatment for this rapidly progressive and easily metastatic neurofibrosarcoma. Photodynamic therapy (PDT) has been developed as an anti-cancer treatment, and 5-aminolevulinic (ALA) mediated PDT (ALA-PDT) has been used to treat cutaneous skin and oral neoplasms. Doxycycline, a tetracycline derivative, can substantially reduce the tumor burden in human and animal models, in addition to its antimicrobial effects. The purpose of this study was to evaluate the effect and to investigate the mechanism of action of combined doxycycline and ALA-PDT treatment of MPNST cells. An 3-(4,5-dimethylthiazol-2-yl)-2,5-diphenyltetrazolium bromide (MTT) assay showed that the combination of ALA-PDT and doxycycline significantly reduce MPNST survival rate, compared to cells treated with each therapy alone. Isobologram analysis showed that the combined treatment had a synergistic effect. The increased cytotoxic activity could be seen by an increase in cellular protoporphyrin IX (PpIX) accumulation. Furthermore, we found that the higher retention of PpIX was mainly due to increasing ALA uptake, rather than activity changes of the enzymes porphobilinogen deaminase and ferrochelatase. The combined treatment inhibited tumor growth in different tumor cell lines, but not in normal human Schwann cells or fibroblasts. Similarly, a synergistic interaction was also found in cells treated with ALA-PDT combined with minocycline, but not tetracycline. In summary, doxycycline can potentiate the effect of ALA-PDT to kill tumor cells. This increased potency allows for a dose reduction of doxycycline and photodynamic radiation, reducing the occurrence of toxic side effects *in vivo*.

## Introduction

Photodynamic therapy (PDT), a new approach to cancer treatment, uses photosensitizers (PS) and light to generate reactive oxygen species, which can interact with biomolecules and subcellular organelles to interrupt key molecular functions. This photoreaction leads to cell death and destruction of tissues [1]. 5-aminolevulinic acid (ALA) itself is not a photosensitizer; but exogenously applied ALA leads to the increased formation of protoporphyrin IX (PpIX, a photosensitizer) by a series of enzymes in the heme biosynthetic pathway [2]. There are two rate-limiting steps in this pathway. The first is ALA production from succinyl CoA and glycine by ALA synthase, which is regulated by heme via a negative feedback mechanism. The other is the formation of heme via the addition of a ferrous iron to PpIX, which is controlled by the rate-limiting enzyme, ferrochelatase (FC). It has been shown that ALA-induced PpIX accumulation is greater in certain tumor cells due to the reduced activity of ferrochelatase (FC) [3] and higher porphobilinogen deaminase (PBGD) activity [4]. In addition, cancer cells usually have high rate of glycolysis followed by lactic acid fermentation (Warburg effect), resulting in abnormalities of iron metabolism and errors in iron insertion which lead to the enhanced accumulation of PpIX in cancer cells [5].

In the treatment of dermatological diseases, topical administration of ALA induces the accumulation of endogenous PpIX, which triggers a photoreaction after light illumination [6]. ALA-mediated PDT (ALA-PDT) has been successfully used in managing different types of skin conditions, such as Bowen’s disease, actinic keratosis, basal cell carcinoma, and Paget’s disease [7, 8]. ALA-PDT has a number of the advantages including minimal invasiveness, low morbidity, good tolerance, and the ability to repeatedly treat the same location.

Neurofibromatosis type 1 (NF1) is one of the most common hereditary neurological disorders affecting 1 in 3,000–4,000 people in the general population [9]. The dermatological features of NF1 include café-au-lait spots, axillar and groin freckling, and cutaneous or subcutaneous neurofibromas. Plexiform neurofibroma (PNF), a debilitating complication of NF1, can be identified at birth by the presence of abundant cellular matrix, collagen fibers, blood vessels, mast cells, and Schwann cells [10]. The lifetime risk for developing a malignant peripheral nerve sheath tumor (MPNST) in NF1 patients is 8–13% [11], which is usually transformed from the PNF. MPNST is a highly aggressive malignant neoplasm associated with significant morbidity and mortality. The five-year survival rate is only 21–41%, although early detection with radical surgery in combination with chemo- and radiotherapy can sometimes cure the disease [12]. Several obstacles make the treatment of MPNST difficult. First, no radiological features can clearly differentiate PNF from MPNST in various imaging studies. Second, the tumor location, size, and early distant metastasis of MPNST make it difficult to be surgically removed. Third, pathological diagnosis of a MPNST is challenging. Finally, there is no effective chemotherapeutic armamentarium. Therefore, it is critical to identify and develop new treatment strategies to tackle this disease.

Hamdoon *et al*. reported the first case using photodynamic therapy to treat neurofibromas [13]. The patient had a large solitary tumor on the neck, which caused refractory pain, dysphagia, and breathing problems. The patient was offered an ultrasound-guided PDT using mTHPC (meta-tetrahydroxyphenylchlorin) as the PS. After PDT treatment, the tumor shrank significantly, which resulted in symptom control for the patient with no morbidity. Recently, a clinical trial using ALA-PDT to treat benign dermal neurofibromas was performed to assess the safety and efficacy of PDT in NF1 tumors on the skin (NCT01682811, http://clinicaltrials.gov/ct2/show/NCT01682811). However, the result is not available yet.

Tetracyclines are broad-spectrum antibiotics, which interfere with protein synthesis by restrict the binding of aminoacyl t-RNA to 30S ribosomes [14]. In addition to their antimicrobial effect, tetracyclines are capable of causing defects in mitochondrial protein synthesis in tumor cells [15]. Studies have shown that doxycycline, a tetracycline derivative, shows potency in inhibiting cancer cell growth and metastases [16–23]. However, doxycycline treatment has not been tested in MPNST. In this study, we explored the cytotoxicity of combined ALA-PDT and doxycycline treatment on MPNST derived cells and found the combined treatment to have a synergistic tumoricidal effect.

## Material and methods

### Cell culture

Several cell lines from different tissues were collected. The S462 cell line, from a malignant peripheral nerve sheath tumor of a patient with neurofibromatosis type 1, was gifted from Nancy Ratner, University of Cincinnati. The human malignant melanoma cell, A375, human lung carcinoma cell, A549, and the mouse colon carcinoma, C26, were purchased commercially. Human lung adenocarcinoma CL1-0 and CL1-5 were derived from poorly differentiated adenocarcinomas [24]. Two human primary cells, Schwann cells (NSC), and fibroblasts (Hs68), as well as mouse fibroblasts (NIH3T3) were collected as normal controls. The primary Schwann cells were purchased from ScienCell Research Laboratories (ScienCell Research Laboratories, Inc. Carlsbad, CA). All cells were cultured in Dulbecco’s modified Eagle’s medium (DMEM) or Roswell Park Memorial Institute medium (RPMI) with 10% fetal bovine serum (FBS) at 37°C with 5% CO_2_.

### ALA-PDT

For ALA treatment, the cells were incubated with ALA (Sigma-Aldrich, MO, USA) at different concentrations in medium containing 10% FBS and phenol red. After incubation for a certain period of time, ALA was removed by rinsing with phosphate buffer solution (PBS). The cells in phenol red-free medium were subjected to light irradiation by using a 635 nm diode laser with a power density of 30 mW/cm^2^.

### Drug treatment (tetracycline, doxycycline and minocycline)

After rinsing with PBS, cells were incubated with different concentrations of doxycycline (Sigma-Aldrich, MO, USA) for 24 h. The cells were then harvested for further MTT analysis. To evaluate the effect of tetracycline and its derivatives, a dose of 50 μg/mL tetracycline, doxycycline, or minocycline was used.

### Analysis of the cause of cell death

To determine the mechanism of cell death caused by ALA-PDT and doxycycline treatments, inhibition of both apoptosis and autophagy were tested. Benzyloxycarbonyl-Val-Ala-Asp-fluoromethylketone (Z-VAD-FMK) is a strong pan-caspase inhibitor that can bind the catalytic domain of caspase, a vital component of the apoptotic cascade, to block the signal transduction irreversibly. Cells were pre-treated with different concentrations of Z-VAD-FMK one hour before treatment with ALA-PDT alone or in combination with doxycycline. To evaluate involvement of the autophagy pathway in the cell death mechanism, 3-methyladenine (3-MA) was employed. 3-MA can inhibit the activity of Vps34 in class III PI3K complexes, and prevent the downstream cascade to form an autophagosome. Cells were pre-treated with 3-MA 30 min before the start of ALA-PDT treatment.

### Immunocytochemistry using Hoechst 33342, propidium iodide (PI) and monodansylcadaverine (MDC) stains

Cells were plated onto cover slips and cultured in a 3.5 cm dish. Twenty-four hours after ALA-PDT and/or doxycycline treatments, cells were stained with Hoechst 33342 (10 μg/mL), PI, 100 μg/mL, and MDC, 0.05 mM at 37°C for 30 min. The cells were then sealed for observation under a fluorescent microscope. To observe the staining of Hoechst 33342 and MDC, the wavelength (λ) used was 330–385 nm, while 510–550 nm was used for PI staining.

### Assays to determine intracellular ALA levels

After treatment with ALA, the cells were washed twice with PBS. Then, 50 μL of ice-cold cell lysis buffer (20 mM Tris-HCl, 150 mM NaCl, 1 mM ethylenediaminetetraacetic acid (EDTA), 1 mM ethylene glycol tetraacetic acid (EGTA), 25 mM β–glycerophosphate, 10% glycerol (v/v), 1% Triton X-100 (v/v), 5 μg/mL leupeptin, 5 μg/mL aprotonin, 2 mM Na_3_VO_4_, 1 mM phenylmethane sulfonyl fluoride (PMSF), and 1 mM dithiothreitol (DTT)) was added to the cells. After 10 min, the cells were collected in an Eppendorf tube followed by centrifugation at 1000× *g* for 10 min. The supernatant of cell lysate was stored at -20°C until use. For chemical derivatization, 1400 μL of acetylacetone reagent (distilled deionized (dd) water:absolute ethanol:acetylacetone = 55:35:15 (v/v)) and 180 μL of 10% formaldehyde were added into 20 μL of cell lysate. After thorough mixing, it was incubated in a water bath (100°C) for 10 min and subsequently cooled on ice. The ALA derivatization complex (1 mL) was subjected to high-performance liquid chromatography (HPLC, Shimadzu corporation, Kyoto, Japan) analysis [25]. The analysis was performed at room temperature at a flow rate of 1 mL/min. The mobile phase contained methanol:dd water:acetic acid = 60:40:0.1 (v/v) and the stationary phase contained octaDecyl-ODS (C_18_). The wavelengths for excitation/emission were 370/460 nm, respectively. Calibration curves were obtained using 0.25, 0.4, 0.5, 1, 2, and 4 μg/mL of ALA in cell lysate. Linear regression analysis was employed to evaluate the linearity, which was calculated by the least square regression method. The measurement of ALA content (μg) was obtained by interpolation. The intracellular uptake of ALA was calculated using the formula:

Intake level (μg/mg protein) = intracellular ALA content (μg) /total protein content (mg).

### Uptake of intracellular chlorin e6 (Ce6)

S462 cells were incubated with complete medium containing 1 mL of 4 μg/mL Ce6 with different concentrations of doxycycline (0, 0.2, 5, and 50 μg/mL). After 4 and 24 hours of incubation, the cells were lysed with 0.1 N NaOH. A portion of the cell lysate was subjected to fluorescent spectrometry (FluoroMax-4 Spectrofluorometer, Horiba Jobin Yvon Inc., Edison, NJ, USA) to measure the content of Ce6. The excitation wavelength was λ_ex_ = 400 nm and emission was λ_em_ = 663 nm. The rest of the cell lysate was subjected to a protein quantitation assay. The amount of Ce6 intake was calculated according to the formula:

$$\text{Intake level }(\text{c}.\text{p}.\text{s}.\;\times \;10^{6}/\text{mg protein})\;=\text{ fluorescence intensity of Ce}6\text{ in cell lysate }(\text{c}.\text{p}.\text{s}.\;\times \;10^{6})\;/\text{ total protein content of lysate }(\text{mg}).$$

### Fluorometric quantification of PpIX

Cell suspension was prepared and treated with ALA for a certain time period. After washing with PBS twice, the cells were re-suspended in 1X Trypsin-EDTA. The mixture was centrifuged and the cells were again re-suspended with 1 mL of ice-cold PBS and transferred to a Falcon tube. The suspension was then analyzed via flow cytometry (BD FACSCalibur^™^ Flow Cytometer, BD Biosciences, Sparks, MD, USA). The fluorescence excitation wavelength was set at 488 nm and the emission wavelength at 605–635 nm, in the FL3 range. The average fluorescence measured among 10,000 cells was defined as the quantity of the intracellular PpIX. The intracellular PpIX accumulation level was obtained using the formula:

$$\text{Intracellular PpIX accumulation }\left(\%\text{ of control}\right)\;=\lambda_{\text{FL}3\;\left(\text{treated}\right)}/\lambda_{\text{FL}3\;\left(\text{control}\right)}\times \;100\%.$$

To observe the fluorescent images of PpIX accumulation intracellularly, the cells were washed and plated, followed by sealing with cover slips. They were then observed using fluorescent microscopy with an excitation wavelength of 510–550 nm.

### Enzymatic activity assay for PBGD and FC

After treatment, the cells were lysed and scratched from the culture dish followed by centrifuge at 12000× *g* for 10 min at 4°C. The supernatant was collected for enzyme analysis. To assess the enzyme activity of PBGD, cell lysate (40 μL) and substrate (10 μL of 5 mM porphobilinogen (PBG)) were mixed at 45°C for 30 min. To terminate the enzyme reaction, 200 μL of ethyl acetate/acetic acid (3:1, v/v) was added to the reaction mixture. After centrifuging at 6000 rpm for 10 min, the product was extracted from the organic phase. The light-induced reaction was performed under exposure to ambient light at room temperature for 15 min. Supernatant (160 μL) was then mixed with 100 μL of 0.5 M HCl followed by centrifuging at 6000 rpm for 10 min. Dd water (500 μL) was used to dilute 100 μL of the lower phase. The mixture was then subjected to the fluorescent spectrometry to measure the enzyme activity of PBGD with excitation and emissions set to 405 and 603 nm, respectively.

To determine the activity of FC the hematoporphyrin (Hp) reagent was prepared, (1 mM Hp, 10 mM sodium palmitate, 10% Tween 20 (w/v), and 1 M Tris-HCl buffer at pH 8.0). Reaction mixture (200 μL) consisted of 10 μL cell lysate, 180 μL Hp reagent and 10 μL 2 mM zinc acetate. The enzyme reaction was carried out at 37°C for 1 h, which was terminated by adding 500 μL of dimethyl sulfoxide (DMSO)/methanol with 0.1 mM EDTA. After centrifugation at 12500 rpm for 10 min at 4°C, 600 μL of supernatant was assayed via fluorescence spectrometry to measure the enzyme activity of FC with excitation and emission wavelengths set to 410 and 580 nm, respectively.

### Statistical analyses

All the data presented are the mean from at least three independent experiments and expressed as mean ± standard deviation. For two group comparisons, the unpaired student’s *t*–test was employed, while one-way analysis of variance (ANOVA) was used for three or more groups. The significance was defined at *p*-value < 0.05.

Before analysis with isobologram [26], the IC_50_ for each of the two treatments (A and B) was identified (IC_50, A_ and IC_50, B_, respectively). The IC_50_ for treatment (A) under treatment (B) was also assessed (IC_50, A/B_). Then, the fractional IC_50_ for treatment A could be obtained by dividing the IC_50, A/B_/IC_50, A_. Likewise, the fraction of IC_50_ for treatment B was IC_50, B/A_/IC_50, B_. Then, the coordinate points represented by the fractional IC_50_ were depicted on the Cartesian plane. The diagonal line on an isobologram represented an additive effect of these two treatments. The points well below the diagonal line represented a synergistic effect between them. Points above the additive diagonal line suggested an antagonistic effect [26, 27].

## Results

### S462 cell growth is suppressed after ALA-PDT and doxycycline treatments

**Fig 1:**
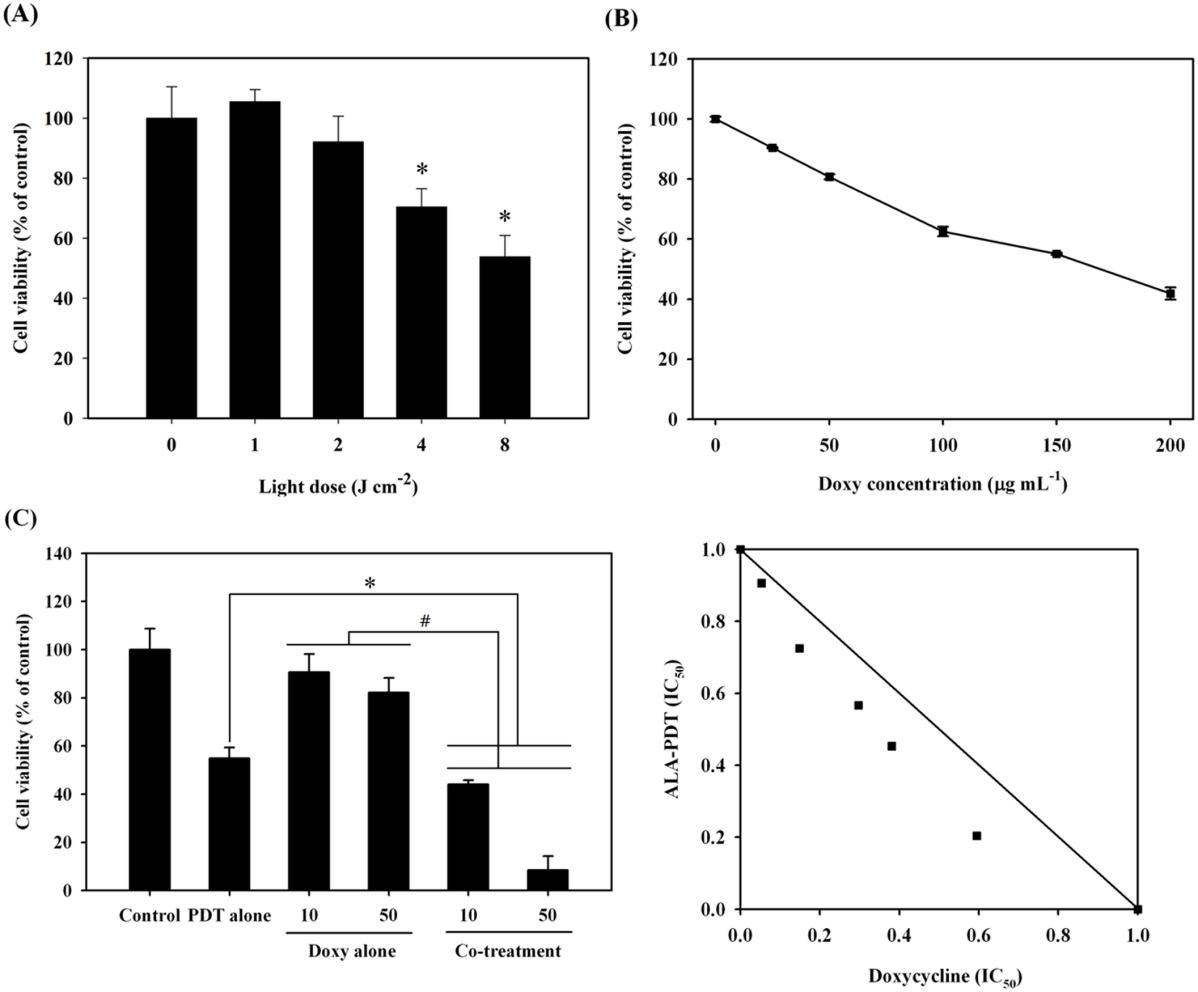
Cell viability of malignant peripheral nerve sheath tumor cell line, S462 under the treatment of 5-aminolevulinic acid-mediated photodynamic therapy (ALA-PDT) alone or in combination with doxycycline. (A), the percentage of S462 cell viability post the treatment of ALA-PDT. The cells were incubated with 1 mM ALA for 24 hours followed by light illumination. The MTT assay was performed to evaluate the cell viability at different light doses. (B), the percentage of S462 cell viability after the treatment of different concentration of doxycycline. (C) Left panel shows the percentage of cell viability under PDT alone and in combination with doxycycline (10- and 50- μg/mL) under the light dose of 8 J/cm^2^. The contour connecting every point (d1, d2) in the isobologram bows inwardly, which indicates a Loewe synergy between the treatment of ALA-PDT and doxycycline (right panel).

To address the feasibility of ALA-PDT for treating MPNST, we first examined the effectiveness of ALA-PDT against the MPNST derived cell line, S462. The cells were treated with 1 mM ALA for 24 h and then irradiated with various doses of light. As shown in Fig 1A, the viability of S462 cells was significantly reduced in a light-dose dependent manner. The LD_50_ light dose was estimated to be approximately around 8.5 J/cm^2^, which was then used for the following study.

We then assessed whether doxycycline could inhibit the growth of S462 cells. As shown in Fig 1B, treatment with doxycycline for 24 h suppressed cell growth in a concentration-dependent manner. The doxycycline LD_50_ for cell growth inhibition was approximately 172.1 μg/mL.

While both of these treatments demonstrated an inhibitory effect on the growth of MPNST cells, the combination of ALA-PDT and doxycycline showed a much stronger cytotoxicity. ALA-PDT treatment alone under a light dose of 8 J/cm^2^ resulted in a cell viability of 54.9 ± 4.5%; however, in combination with doxycycline treatment (10 μg/mL), survival was significantly reduced to 44.0 ± 1.8%. More strikingly, cell viability decreased to 8.5 ± 5.7% of the control when the cells were treated with ALA-PDT and 50 μg/mL of doxycycline (left panel of Fig 1C). The isobologram analysis between ALA-PDT and doxycycline treatments showed a synergistic effect, since the coordinate location in the isobologram Cartesian plane is below the additivity line (right panel of Fig 1C).

### The mechanisms of cell death caused by the combined treatment of ALA-PDT and doxycycline

**Fig 2:**
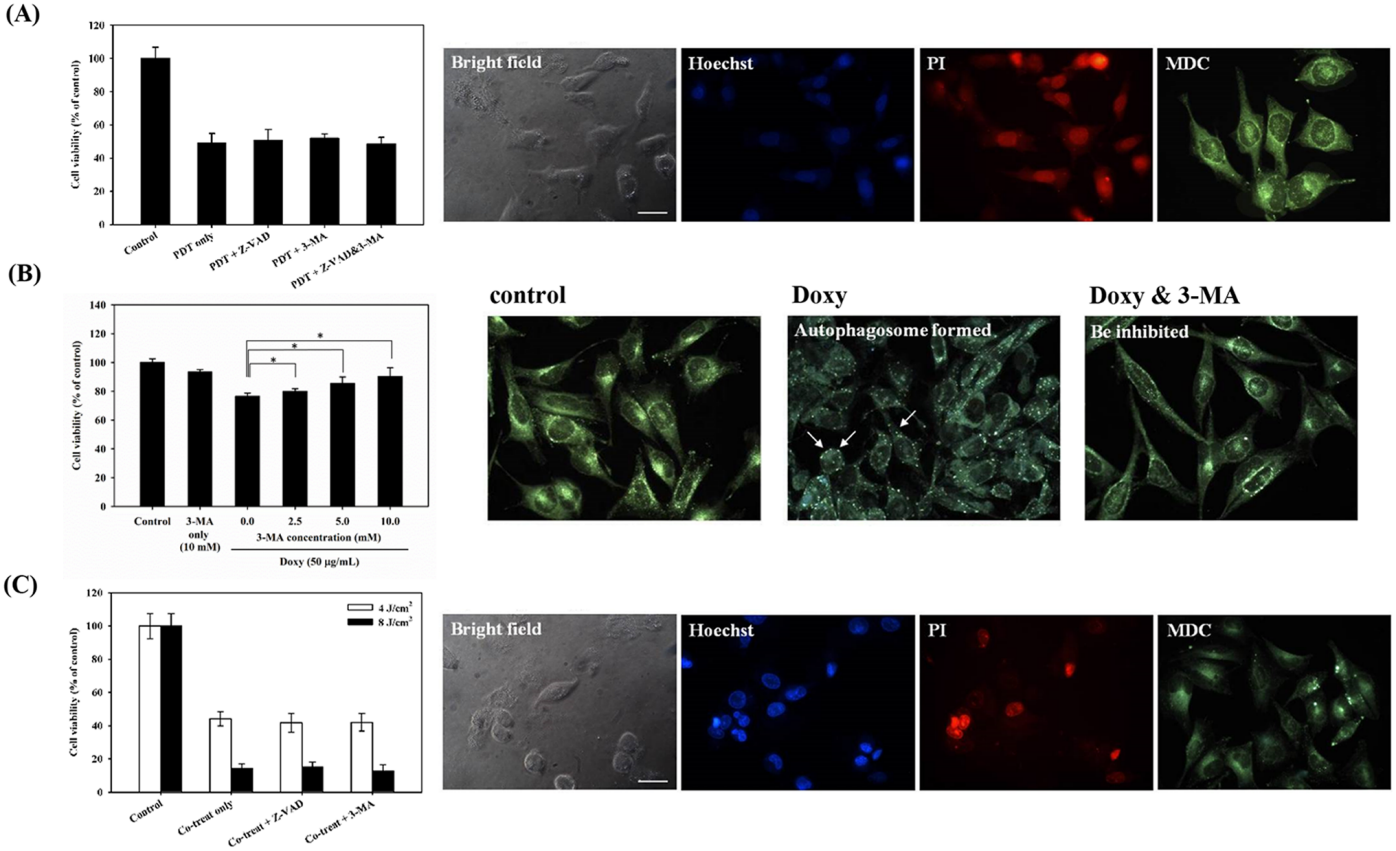
Cell viability and death mode under the treatment of ALA-PDT, doxycycline, and combined treatment ALA-PDT/doxycycline. (A) The percentage of cell viability treated with ALA-PDT did not have a significant change after the pretreatment of benzyloxycarbony-Val-Ala-Asp-fluoromethylketone (co-treat + Z-VAD) or 3-methyladenine (co-treat + 3-MA) (left panel). The morphology and fluorescence staining of S462 cells were observed by bright field and fluorescent microscopy after ALA-PDT (right panel) (B) The survival rate of S462 cell was rescued when pre-treated with different concentrations (0, 2.5, 5.0, and 10.0 mM) of 3-MA (left panel). The right panel shows the results of fluorescent microscopical analysis. Abundant autophagosomes stained by MDC (bright punctate in cytoplasm) were found in the cytoplasm after treatment of doxycycline (doxycycline treatment); however, after co-treatment of 3-MA (doxycycline + 3-MA), the amount was decreased conspicuously. (C) The percentage of cell viability received ALA-PDT/doxycycline did not have a significant change under the pretreatment of Z-VAD (co-treat + Z-VAD) or 3-MA (co-treat + 3-MA) (left panel). The morphology of the cells post the co-treatment was observed by bright field and fluorescent microscopy (right panel of (C)).

Both apoptotic and autophagic cell deaths have been found in ALA-PDT treated cells [28, 29]. We treated the cells with caspase inhibitor Z-VAD-FMK and autophagy inhibitor 3-MA to understand the mode of cell death in S462 cells. As shown in the left panel of Fig 2A, pretreatment with either Z-VAD-FMK or 3-MA did not increase the viability of cells treated with ALA-PDT, suggesting the cell death is not through apoptosis or autophagy in PDT-treated S462 cells. Microscopic observation in a bright field revealed the rupture of the plasma membrane in cells treated with ALA-PDT (right panel of Fig 2A). In addition, fluorescent microscopy showed significant PI staining in cells after PDT treatment, indicating that ALA-PDT mainly results in necrotic cell death in S462 cells. Indeed, staining with Hoechst 33342 did not show chromatin condensation, although there were a few autophagosomes seen with MDC staining after ALA-PDT treatment.

Cell viability decreased to approximately 78% in S462 cells treated with 50 μg/mL of doxycycline, which could be rescued by 3-MA in a dose-dependent manner (left panel of Fig 2B). Microscopic observations showed a significant increase of autophagosomes stained with MDC in the cytoplasm of S462 cells treated with doxycycline. The staining was lowered with 3-MA pre-treatment (right panel of Fig 2B). These findings suggest doxycycline induces autophagic cell death in S462 cells.

Combined treatment with ALA-PDT/doxycycline caused a further lowering of cell viability compared to the ALA-PDT alone, at a light energy of 4 or 8 J/cm^2^ (Co-treat only, left panel of Fig 2C). However, pretreatment with either Z-VAD-FMK or 3-MA could not rescue the cell viability after the combined treatment. Staining with PI allowed the cells to be observed easily via fluorescent microcopy (right panel of Fig 2C). A near-total absence of chromatin condensation was observed in the cells with Hoechst 33342 staining, but few formations of autophagosome were seen after staining with MDC (right panel of Fig 2C). Taken together, these findings indicate that the combined treatment of ALA-PDT/doxycycline results in cell death primarily through necrosis rather than apoptosis or autophagy.

### Doxycycline potentiated the ALA-PDT effect through the increase of intracellular PpIX and uptake of ALA

**Fig 3:**
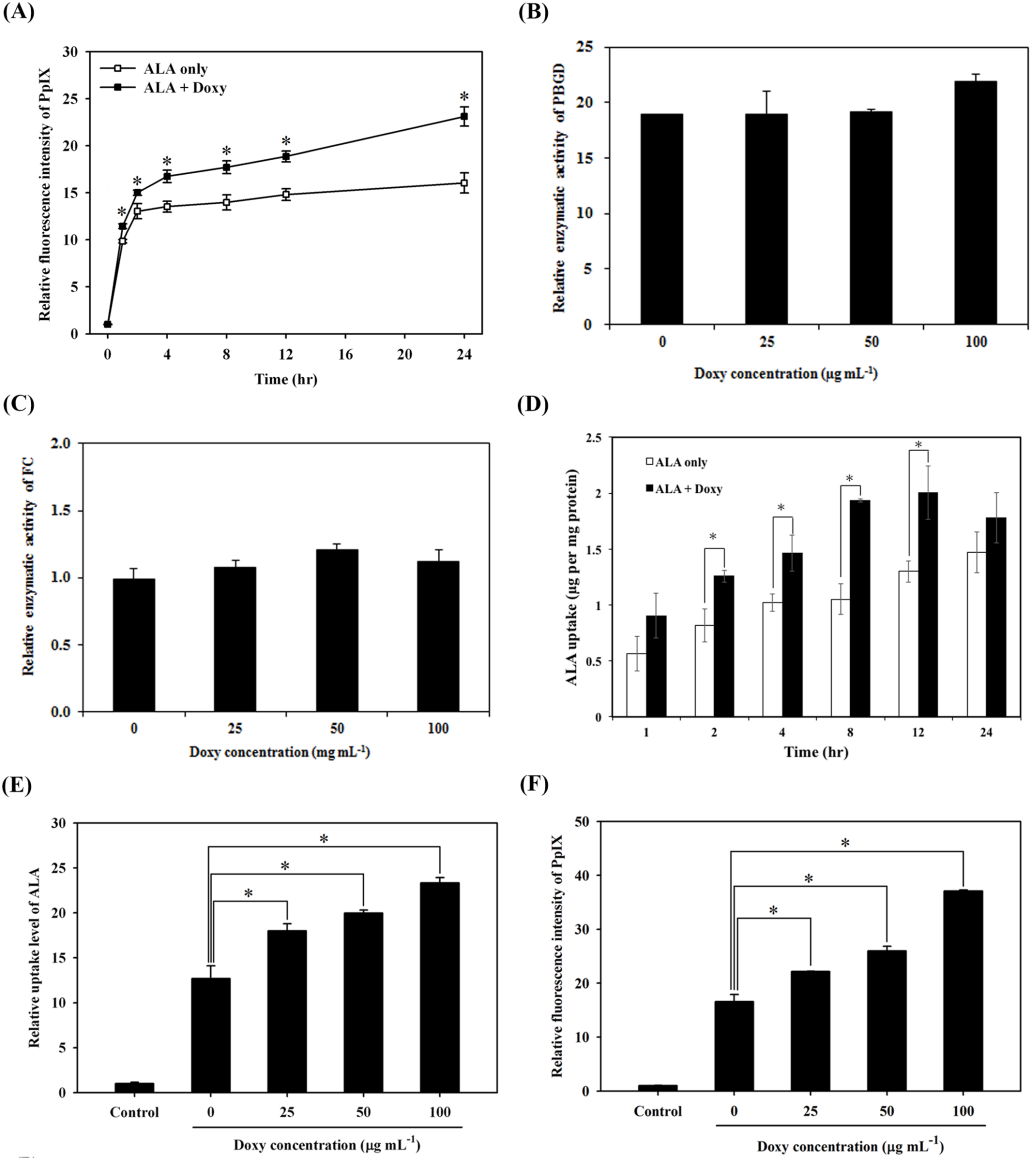
Accumulation of protoporphyrin IX (PpIX), enzyme activities for porphobilinogen deaminase (PBGD) and ferrochelatase (FC), and uptake of 5-aminolevulinic acid (ALA) in cells co-treated with ALA and doxycycline. (A) The relative fluorescence intensity of PpIX was evaluated against the time post either ALA (open square) or the combined treatment of ALA and doxycycline (filled square). The relatively enzyme activity of PBGD (B) and FC (C) after the combined treatment of ALA and different concentration of doxycycline. (D) The uptake of ALA was assessed against the time post either ALA alone (open square) or the combined treatment of ALA and doxycycline (filled square). (E and F) The relative uptake level of ALA (E) and the fluorescence intensity of PpIX (F) were measured in S462 cells after incubated with 1 mM of ALA and different concentrations of doxycycline for 24 hours (*, p<0.05).

To elucidate the mechanism involved in the increased cytotoxicity caused by the combined ALA-PDT/doxycycline treatment, we examined whether the combined treatment caused an increase in PpIX levels in the cells. As shown in Fig 3A, co-treatment with ALA and doxycycline significantly increased intracellular PpIX concentrations compared with cells treated with ALA alone, suggesting the increased cytotoxicity may be associated with the increased cellular PpIX levels.

In the ALA-PpIX conversion pathway, the increased accumulation of PpIX can be due to the activation PBGD in the cytosol or to the inactivation of FC in mitochondria [30, 31]. We did not observe any difference in enzyme activities of PBGD (Fig 3B) or FC (Fig 3C) among S462 cells treated with either ALA-PDT alone or in combination with different concentration of doxycycline (25, 50, or 100 μg/mL). Meanwhile, the ALA uptake significantly increased in a time-dependent manner after the combined treatment (Fig 3D). The ALA uptake and intracellular PpIX accumulation also increased with increasing doxycycline doses in S462 cells, compared to those treated with ALA alone (Fig 3E and 3F).

**Fig 4:**
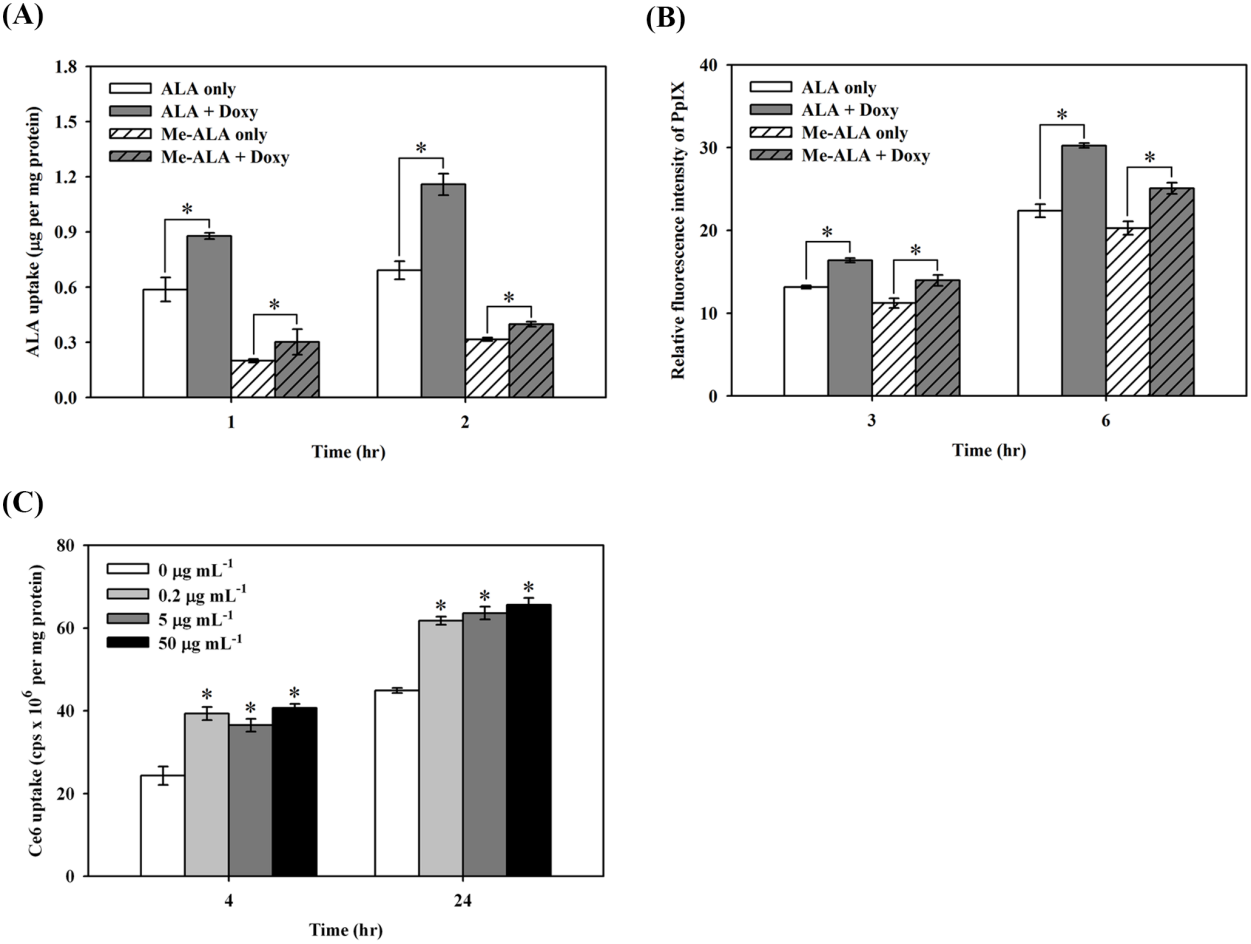
The effect of doxycycline on cellular uptake of methyl 5-aminolevulinic acid (me-ALA) and chlorin e6 (Ce6). (A and B) Application of me-ALA leads to the uptake of ALA and accumulation of PpIX, similar to that found after ALA treatment. The duration of incubation was one or two hours for the uptake assay (A). For PpIX accumulation measurements, these four groups of cells were incubated for three and six hours (B). (C) The increased uptake of Ce6 was also found by doxycycline. *, p<0.05).

To examine whether the effect of doxycycline on photosensitizer uptake is ALA specific, we tested two different photosensitizers, methyl 5-aminolevulinic acid (me-ALA) and Ce6. As shown in Fig 4A, the uptake of me-ALA significantly increased after incubation with doxycycline for one or two hours. Similarly, PpIX levels also increased in those samples post-treatment for either three or six hours (Fig 4B). Likewise, Ce6 uptake in S462 cells also increased in time- and dose-dependent manners when co-incubated with doxycycline (Fig 4C). These results indicate that doxycycline enhancement of cellular uptake of photosensitizers is not specific to ALA.

### Cell viability, ALA uptake and accumulation of PpIX in tumor and normal cells

**Fig 5:**
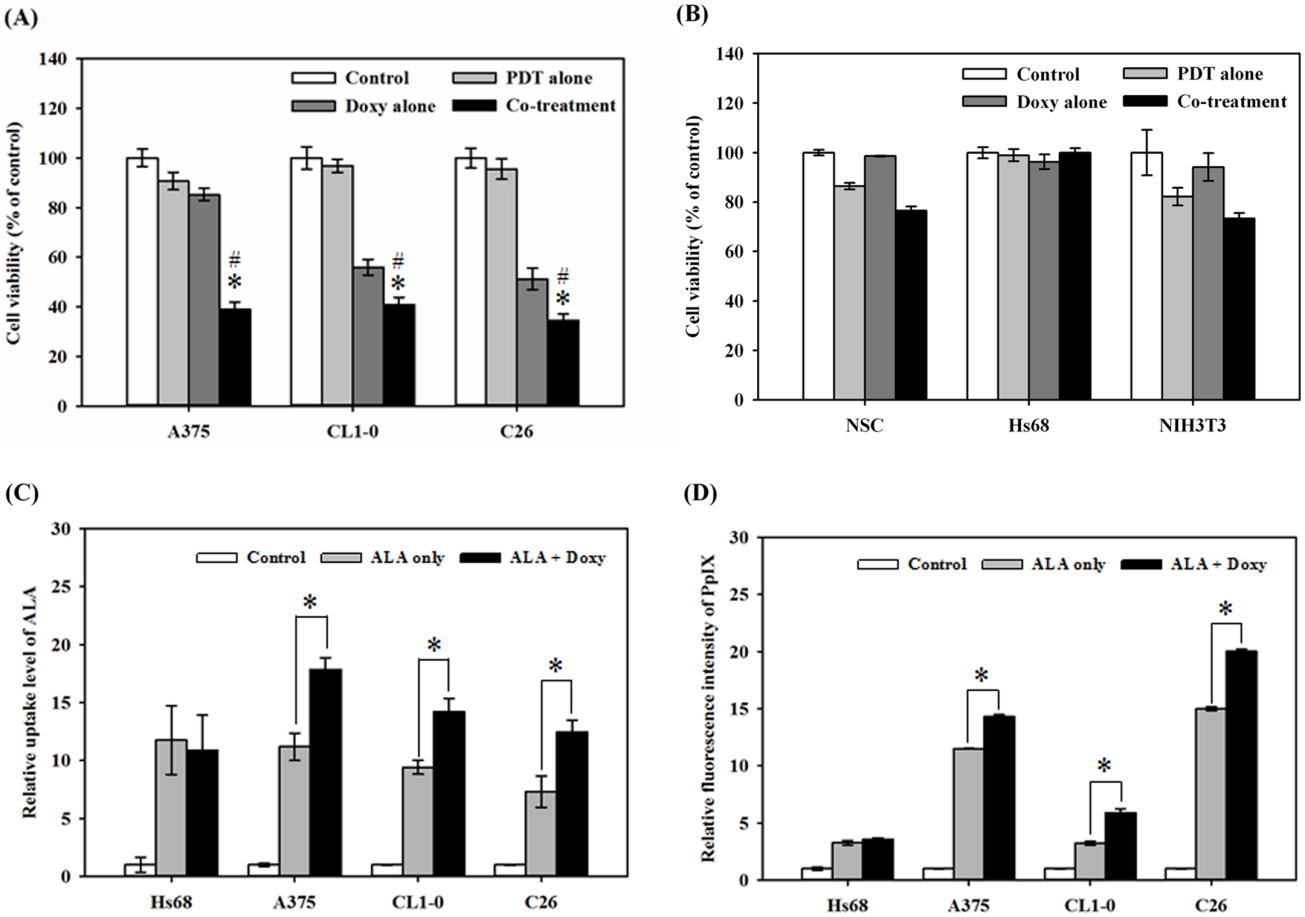
Cell survival, uptake of ALA and accumulation of PpIX in tumor and normal cells. (A), the percentage of cell viability post the treatment of ALA-PDT alone (PDT alone), doxycycline alone (Doxy alone), combined treatment (co-treatment) and sham operation (control) was evaluated in two malignant cell lines (the human malignant melanoma cell, A375 and lung adenocarcinoma CL1-0) and the mouse colon carcinoma, C26. The light dose for A375 cells is 9 J/cm^2^; 12 J/cm^2^ is used for CL1-0 and C26. The combined treatment significantly inhibited the growth of the five different cancer cells. (B), the percentage of cell viability in normal human Schwann cells (HSC), human fibroblast (Hs68) and mouse fibroblast (NIH3T3) cells after the same treatment as shown in (A). The light dose for NSC, Hs68, and NIH3T3 cells is 8 J/cm^2^. There were no significant changes in cell viability after the treatment of PDT (PDT alone), doxycycline (Doxy alone) or in combination (Co-treatment). The uptake of ALA (C) and accumulation of PpIX (D) after the treatment of ALA-PDT only (ALA alone), combined treatment ALA-PDT/doxycycline (ALA + Doxy), or sham operation (control) in both normal human fibroblast (Hs68) and four malignant cell lines (A375, CL1-10, and C26).

Given the efficacy of doxycycline in enhancing ALA uptake and the PDT efficacy in S462 cells, we then evaluated whether this efficacy can be found in other cell types. ALA-PDT/doxycycline synergistic cytotoxicity could also be observed human malignant cell lines (A375 and CL1-0) and mouse colon tumor cells (C26) (Fig 5A). Similar results were also found in A549 and CL1-5 human lung cancer cells (data not shown). On the contrary, the cell viability did not change significantly in normal human Schwann cells, fibroblasts (Hs68), or mouse embryonic fibroblasts (NIH3T3) (Fig 5B). We observed that increased cytotoxicity caused by ALA-PDT/doxycycline combined treatment correlated with the increase of cellular ALA uptake (Fig 5C) and PpIX accumulation (Fig 5D) in malignant cells; however, there were no marked changes in ALA uptake or PpIX levels in normal human Hs68 fibroblast cells.

### Cell survival decreased with increasing ALA uptake and PpIX accumulation in S462 cells under the combined treatment of ALA and tetracycline derivatives

**Fig 6:**
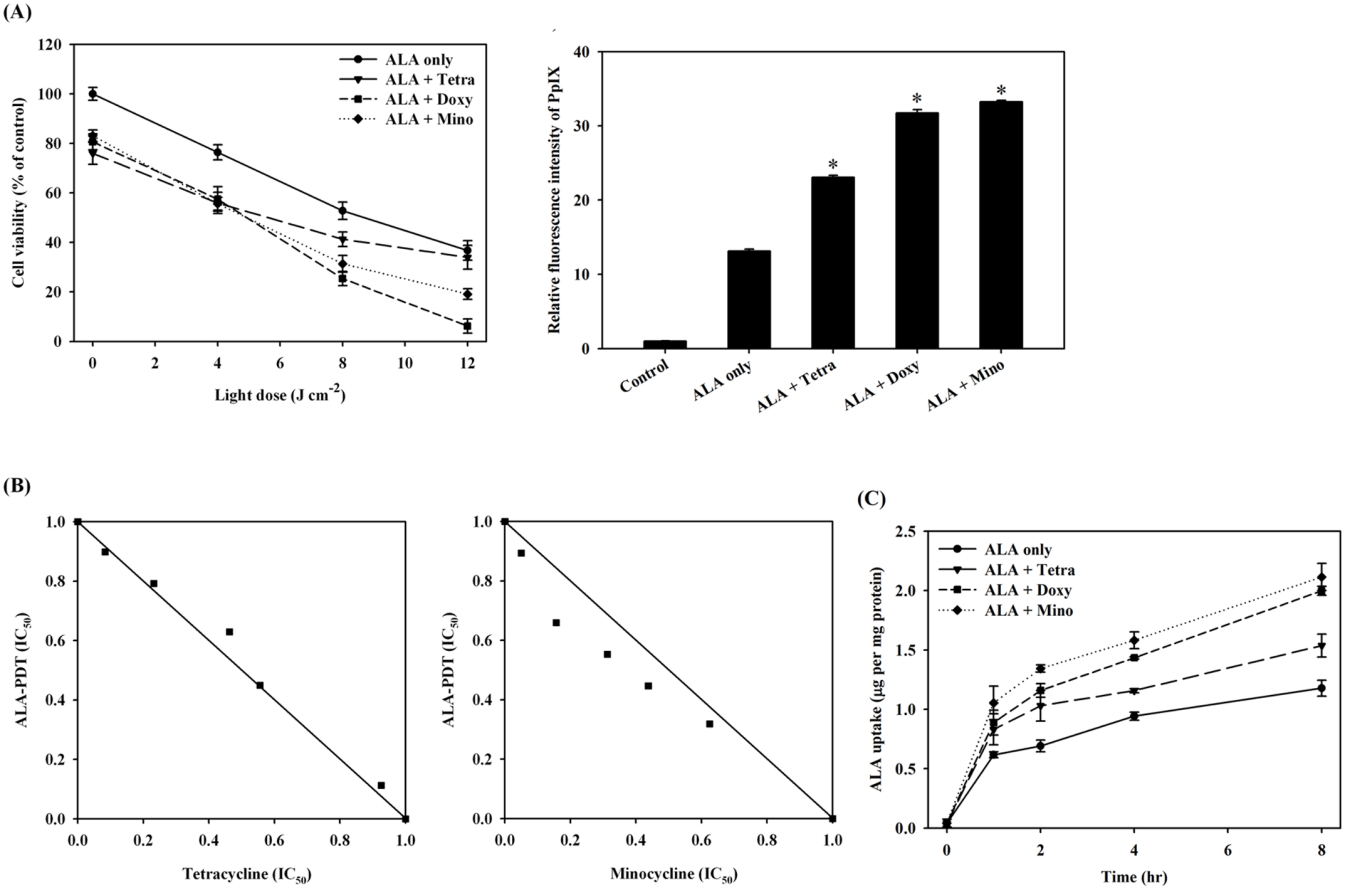
Synergistic effects of tetracycline and its derivatives combined with ALA-PDT treatment. (A), Left panel shows the percentage of cell viability after the treatment of ALA-PDT only (ALA only), or in combination with tetracycline (ALA + Tetra), doxycycline (ALA + Doxy), or minocycline (ALA + Mino). Right panel shows the relative PpIX amounts after ALA only and the combined treatment. (B), isobologram analysis for the combined treatment of ALA-PDT/tetracycline (left panel), and ALA-PDT/minocycline (right panel). (C), the amounts of ALA uptake after the ALA only and the combined treatment.

We next evaluated the presence of a synergistic effect of ALA-PDT with the doxycycline related drugs, tetracycline and minocycline. After the incubation of S462 cells with 1 mM ALA for 24 h in the presence or absence of 50 μg/mL of doxycycline, tetracycline or minocycline, the cells were irradiated with different doses of light. As shown in the left panel of Fig 6A, the light dose that induced 50% cytotoxicity (LD_50_) was approximately 8–9 J/cm^2^ for ALA-PDT only. The LD_50_ for the combined incubation of ALA and tetracycline was approximately 6.2 J/cm^2^; but for ALA and doxycycline or minocycline were approximately 4.9 and 5.1 J/cm^2^, respectively. The PDT efficacy among these three antibiotics correlates with the amounts of intracellular accumulated PpIX (Fig 6A right panel). Isoborogram analysis to test for a synergistic effect showed that the addition of minocycline to ALA-PDT treatment demonstrated a positive synergism (the right panel of Fig 6B), but only an additive effect was seen for the combination of ALA-PDT and tetracycline (Fig 6B, left panel). Fig 6C further demonstrates that the increased uptake of ALA in the presence of doxycycline, tetracycline and minocycline was incubation-time dependent.

### BETA transporters do not increase ALA cellular accumulation in the presence of doxycycline

**Fig 7:**
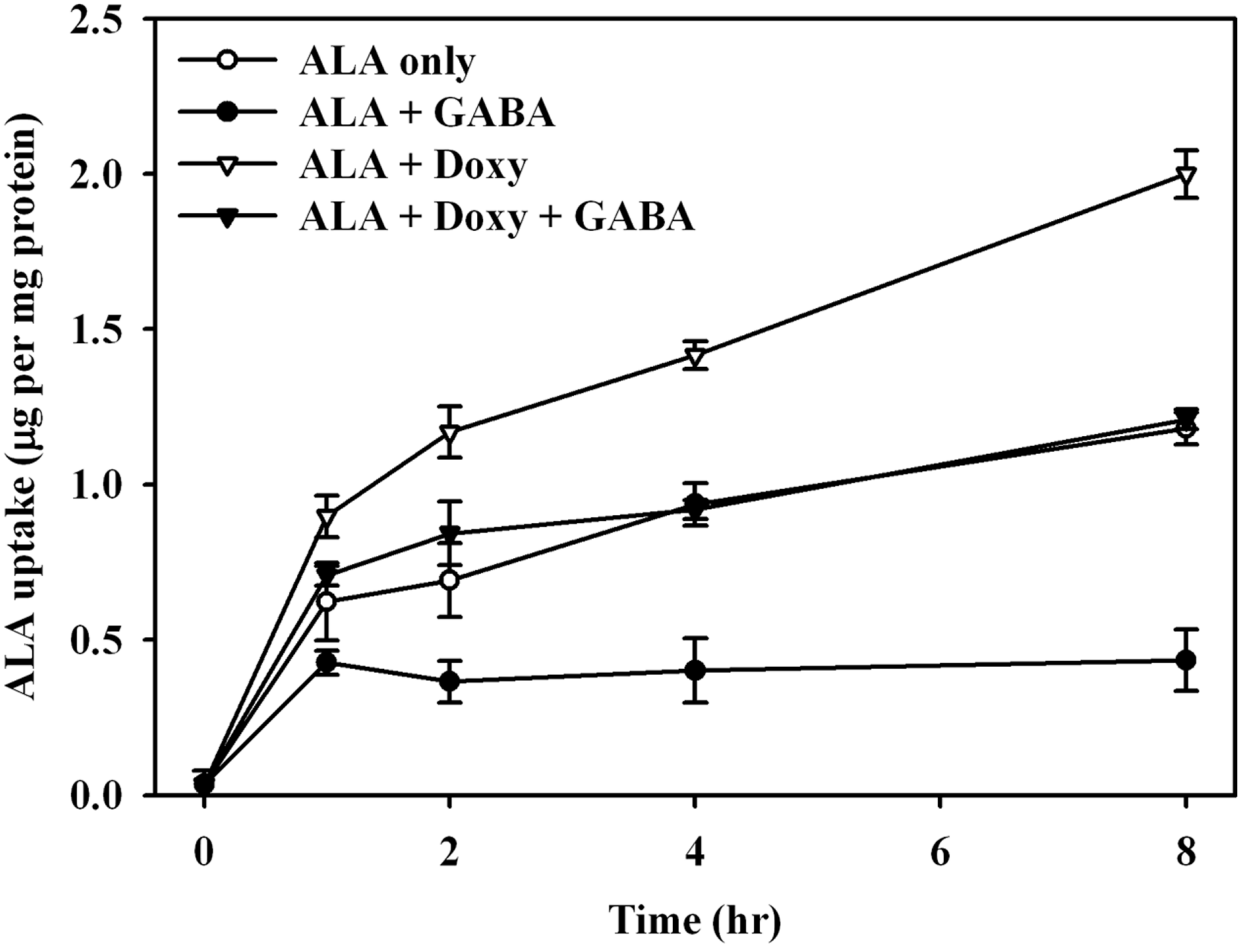
ALA uptake in MPNST, S462 cells with combined treatment of ALA-PDT, doxycycline, and GABA. Measurements of intracellular uptake of ALA were recorded by the time after the treatment of 1 mM of ALA (ALA only), 1 mM of ALA and 10 mM of γ–aminobutyric acid (ALA + GABA), 1 mM of ALA and 50 μg/mL of doxycycline (ALA + Doxy), and 1 mM of ALA, 50 μg/mL of doxycycline and 10 mM of γ–aminobutyric acid (ALA + Doxy + GABA).

Recently, BETA transporters, consisting of GABA (γ-aminobutyric acid) transporters (GAT) 1–3, taurine transporter (TART) and betaine/GABA transporter (BGT-1) [32, 33], have been shown to be involved in ALA and Ce6 uptake [34, 35]. We investigated whether doxycycline treatment would affect BETA transporter activity. We argued that if doxycycline would affect BETA transporter efficiency, the uptake of ALA would be reduced in the presence of excess GABA, since GABA is the substrate of the BETA transporter. As shown in Fig 7, compared to ALA alone, cellular uptake of ALA was significantly suppressed in S462 cells pretreated with GABA. However, when comparing cells treated with GABA (ALA + GABA, Fig 7) and those treated with both GABA and doxycycline (ALA + Doxy + GABA, Fig 7), the increased ALA uptake induced by doxycycline could still be found in cells pre-treated with GABA. These findings suggest that the mechanism for doxycycline-induced increasing ALA uptake might not only depend on the function of the BETA transporter.

## Discussion

Recently, multiple drug regimens have been shown to be more effective to manage or eradicate malignant tissues. In this report, we demonstrated that doxycycline and its derivative could selectively enhance the therapeutic efficacy of ALA-mediated PDT in a MPNST derived cell line, S462. Although treatment with either ALA-PDT or doxycycline alone was moderately effective to suppress the growth of S462 cells, the combined treatment revealed a synergistic cytotoxic effect, as determined by isobologram analysis. In addition, we found that the enhancement of ALA uptake and PpIX accumulation induced by doxycycline is more pronounced in cancer cells compared to normal cells. The combined treatment of topical ALA-PDT and doxycycline given orally was first tried in patients with acne conglobate [36]. Another study using phenothiazine chloride-induced anti-microbial PDT treatment in periodontitis patients with uncontrolled diabetic mellitus showed enhanced effectiveness with the topical application of doxycycline [37].

Both apoptotic and non-apoptotic pathways are involved in cell death associated with PDT, depending on the cell type, the photosensitizer, and the energy dose [38]. Previously, we have shown that ALA-PDT could induce autophagic cell death in PC12 and CL1-0 cells [28]. In this study, we found that necrosis is the main cell death mode in MPNST derived S462 cells when treated with ALA-PDT (Fig 2A). Although autophagic cell death was found in S462 cells treated with doxycycline, cell death caused by the combined treatment of ALA-PDT and doxycycline could not be rescued by the inhibitors of apoptosis and autophagy, Z-VAD-FMK and 3-MA, respectively (Fig 2). Meanwhile, significant PI staining was found in the cells treated with ALA-PDT/doxycycline, suggesting that necrosis is the main cell death mode induced by this combined treatment.

Extracellular ALA can be taken into cellular cytoplasm by passive diffusion or active transport through PEPT-1/2 or BETA transporters [34, 39]. In the cytoplasm, ALA is catalyzed by a series of enzymes (including PBGD) to produce coproporphyrinogen III, which can be hauled into mitochondria through ABCB6 transporters and then catalyzed to yield the photosensitizer, protoporphyrin IX (PpIX). With the addition of iron ion (Fe^2+^) and the catalytic activity of FC, heme can be synthesized. If the iron is chelated, the accumulation of PpIX will increase significantly. A few studies found that the iron-chelators, EDTA and desferrioxamine (DEF) enhanced the accumulation of PpIX in ALA-treated cells [40–42]. More recently, quinolone chemicals including enoxacin, ciprofloxacin, and norfloxacin, were found to potentiate ALA-mediated photodamage due to the increased PpIX accumulation in HeLa [43] and in human meningioma cells [44]. Increase of iron supply by treatment of hemin or ferric-nitrilotriacetate (Fe-NTA) can counteract the effectiveness resulting from the combination treatment with enoxacin. On the contrary, treatment with an inhibitor of heme oxygenase, Sn-protoporphyrin, led to an increase of PpIX accumulation with subsequent photodamage [43]. Our study revealed that after combined treatment, the accumulation of PpIX increased significantly in MPNST cells, which probably resulted from an increase of ALA uptake, rather than the changes of PBGD or FC enzyme activity. Cell viability of S462 cells after PDT treatment alone was around 50% (Fig 1C); nevertheless, it was reduced to only 10% after PDT and 50 μg/mL doxycycline combined treatment. Fig 3A shows that the factor of relative fluorescence intensity of PpIX was approximately 14 without doxycycline; but it increased to approximately 22 after adding doxycycline to the medium. With the combined treatment, the accumulation of PpIX increased about 57%, which can account for the additional cytotoxic effect with light irradiation. Thus, doxycycline enhancement of PpIX accumulation should be a major contributing factor for its potentiation effect on PDT.

Previous studies have suggested that the strong iron-chelating activity in tetracycline and its derivatives, doxycycline and minocycline, was highly correlated to their efficacy [45–47], since the removal of iron by doxycycline treatment might lead to the marked enhancement of PpIX accumulation. This study showed that the iron-chelating activity is not the only explanation, given that doxycycline also increased the cellular uptake of another photosensitizer, Ce6 (Fig 4C).

In MPNST cells, the uptake of ALA resulted in faster PpIX accumulation than the uptake of me-ALA did (Fig 4B). This is similar to a prior report with keratinocytes [48]. In the present study, we found that doxycycline could potentiate this effect more prominently with ALA uptake, and to a lesser extent with me-ALA uptake. The ALA uptake systems have been reported by a few studies. Some authors have postulated that ALA is transferred through the di- and tri-peptide transporters PEPT1 and PEPT2 in pancreas tumor cells and in yeast transfected by intestinal and renal transporters [31, 49]. In cholangiocarcinoma cells, ALA transport was also found to be mediated by the PEPT1 system [50]. But in colon adenocarcinoma cells, Rud *et al*. suggested that BETA transporters are involved in ALA transport [35]. Further studies in mammary adenocarcinoma LM3 cells showed that ALA shared the same transport system with γ–aminobutyric acid (GABA), which could be one of the BETA transporters, GAT-2 [39]. Similar to colon and mammary adenocarcinoma cells, the BETA transporter may be one of the channels for ALA uptake, given that the application of transporter competitor GABA markedly reduced ALA uptake in both ALA-PDT and ALA-PDT/doxycycline treated cells. Further research is needed to validate this phenomenon. We also found that Ce6 uptake could be enhanced by treatment with doxycycline, although the uptake might be through the APOB/E receptor [51], which is increased by the treatment of doxycycline [52].

In this study, we also showed that the enhancement of ALA-PDT by doxycycline with different malignant cell lines and cytotoxicity correlated to the increase of PpIX accumulation and ALA uptake (Fig 5D). However, these effects were not observed in normal Schwann cells, and both human and mouse fibroblasts. These findings suggest that tumor cells are more sensitive to the combined treatment of ALA-PDT with doxycycline than healthy cells.

MPNST, a devastating neurofibrosarcoma associated with NF1, is a difficult disease to treat with current therapeutic options; however, it is particularly suitable for PDT. For example, MPNST is usually located at the body surface where irradiation penetrance is attainable, and the illuminated area can be controlled to accommodate the tumor size and avoid surrounding tissues. Doxycycline, a tetracycline derivative is a well-tolerated antibiotic with good pharmacokinetics properties including nearly 100% oral absorption and a long serum half-life. Moreover, doxycycline is known to effectively cross the blood-brain barrier. Taken together, the combination of ALA-PDT/doxycycline has a good potential for treating cancers like MPNST. Further clinical trials using oral doxycycline with local ALA-PDT treatment will be needed to determine if this strategy confers a good tumoricidal effect for MPNST.

## Supporting information

S1 File Datasets. (ZIP)
